# The Accuracy of Self-Reported Body Weight Is High but Dependent on Recent Weight Change and Negative Affect in Teenage Girls

**DOI:** 10.3390/ijerph17218203

**Published:** 2020-11-06

**Authors:** Corinna Koebnick, Brit Saksvig, Xia Li, Margo Sidell, Tong Tong Wu, Deborah R. Young

**Affiliations:** 1 Department of Research & Evaluation, Kaiser Permanente Southern California, Pasadena, CA 91101, USA; Xia.X.Li@kp.org (X.L.); Margo.A.Sidell@kp.org (M.S.); Deborah.R.Young@kp.org (D.R.Y.); 2 University of Maryland School of Public Health, College Park, MD 20742, USA; bsaksvig@umd.edu; 3 Department of Biostatistics and Computational Biology, University of Rochester, Rochester, NY 14642, USA; Tongtong_Wu@URMC.Rochester.edu

**Keywords:** body mass index, obesity, validation, self-report, adolescence

## Abstract

Background: Research studies often rely on self-reported weight to calculate body mass index. The present study investigated how the accuracy of self-reported body weight in adolescent girls is affected by overweight/obesity, race/ethnicity, and mental health factors. Methods: In a cohort of girls who participated in the Trial of Activity for Adolescent Girls at ages 11 and 17 (*n* = 588), self-reported and measured weight were compared, and linear regression models were fitted to model the over- or underreporting. The Center for Epidemiological Studies-Depression Scale (CES-D) was used to calculate depressive symptom subscales for negative affect, anhedonia and somatic symptoms. Results: Allowing 3% difference between self-reported and measured weight for the correct reporting of body weight, 59.2% of girls reported their weight correctly, 30.3% underreported (−5.8 ± 4.8 kg), and 10.5% overreported (4.3 ± 3.5 kg). The average difference between self-reported and measured body weight was −1.5 ± 4.3 kg (*p* < 0.001). Factors for misreporting body weight were overweight (β ± SE − 2.60 ± 0.66%), obesity (β ± SE − 2.41 ± 0.71%), weight change between ages 11 and 17 (β ± SE − 0.35 ± 0.04% for each kg), height change between ages 11 and 17 (β ± SE 0.29 ± 0.10% for each cm), and negative affect (β ± SE − 0.18 ± 0.08% for each score unit). Conclusions: The difference between self-reported and measured body weight in adolescent girls is relatively small. However, the accuracy of self-reported body weight may be lower in girls with overweight or obesity, recent weight and height change, and higher negative affect.

## 1. Introduction

In epidemiologic studies but also in clinical settings, measuring weight and height directly is not always possible or convenient. Hence, these settings often rely on self-reported weight and height to calculate body mass index (BMI) [1,2,3]. The demand for self-reported data can be expected to increase due to the use of web-based data assessments [3] and changing needs for research during an ongoing pandemic [4].

Generally, the precision (random errors) and accuracy (systematic errors) of BMI can affect the ability of a study to predict health outcomes. Although measured and self-reported BMI are highly correlated, self-reported BMI may be systematically biased [5,6,7,8]. Based on National Health and Nutrition Examination Survey data (NHANES), the misclassification of individuals who were underweight and obese ranged from 30–40% [9]. Understanding the accuracy of self-reported vs. measured body weight is particularly important considering the social pressure of a preference for thinness in adolescent girls. Based on previous studies in adolescent girls, errors in reporting body weight are a bigger concern than in reporting height [10]. Women and younger adults tend to underreport their weight more than men and older adults while underreporting of height in women is mostly negligible [5]. In Western cultures, the self-perception of physical appearance is an essential factor for adolescents’ self-esteem, especially in girls [11,12]. This problem is less pronounced in boys, as an achievement of the ideal body form for men is not related to being thin, but to an increase in muscularity [13].

The accuracy of self-reported weight in adolescent girls varies [10,14]. A literature review showed that self-reported data underestimated overweight prevalence, and that one-fourth to one-half of those overweight would be missed when relying on self-report [10]. Previous studies have indicated that underreporting in adolescent girls increased with age and was highest in young adulthood [15]. Most studies explaining the accuracy of self-reported body weight in adolescent girls have focused on factors such as obesity, socioeconomic status, physical activity level, and race/ethnicity [10,15,16,17]. Some studies indicate that the accuracy of self-reported weight may also be affected by an adolescent’s body perception and dissatisfaction [18,19,20]. Body dissatisfaction is frequent in adolescents and associated with obesity and depressive symptoms [21]. Given the wide range of accuracy levels observed in previous studies [10], body perception as well as depressive symptoms may explain some of the observed variation in the accuracy of self-reported weight beyond sociodemographic factors.

The present study investigated the association between accuracy of self-reported weight in adolescent girls, the presence of overweight and obesity, recent weight changes, physical self-description, and depressive symptoms such as negative affect (strong experience of negative emotions such as anger, contempt, and anxiety), anhedonia (the diminished capacity to experience pleasure), and somatic symptoms (experience of appetite changes, lack of energy, sleep disturbance, and general aches and pains). This knowledge may help future studies relying on self-reported weight in adolescents to adjust for factors predicting measurement accuracy to better estimate health risks related to overweight and obesity in this population.

## 2. Materials and Methods

### 2.1. Design

For the present study, we analyzed a cohort of girls who participated in the University of Maryland (MD, USA) field site of the Trial of Activity for Adolescent Girls (TAAG 1) at 11 years of age (8th grade in 2006) and TAAG 2, a follow up of University of Maryland field site TAAG 1 with participants at 17 years of age (11th grade in 2009). TAAG has been previously described [22]. Briefly, it was a multicenter group-randomized trial designed to test an intervention to reduce the usual decline in moderate to vigorous physical activity in middle school girls and included six field centers [23]. Three years after the TAAG intervention (TAAG 1), 730 participants from the Maryland field center were invited for a follow-up study (TAAG 2), of which 588 agreed and were remeasured [24]. For the present study, we included data from girls who participated in both TAAG 1 and TAAG 2 as our final analytical study group (*n* = 588 girls). Details about the TAAG 1 and TAAG 2 are discussed elsewhere [25]. Briefly, the characteristics of the two cohorts are not statistically significantly different but TAAG 2 girls had families with slightly less low income and slightly more likely to be obese [25]. The study is currently approved by the Internal Review Board of Kaiser Permanente Southern California (IRB # 10105, approved on 7 August 2013).

### 2.2. Measures

#### 2.2.1. Weight and Height

TAAG 1 and 2 data collectors measured height and weight after the girls removed their shoes and heavy clothing. Body weight was measured to the nearest 0.1 kg with a calibrated scale. Weight and height were measured three times, and the average of the 3 measures was calculated. BMI was calculated as average weight (kg) divided by average height squared (m^2^) [25]. Height was measured to the nearest mm using a Shorr measuring board. TAAG 2 girls also self-reported their weight and height as part of the survey administered on the visit day before their weight and height was directly measured.

Based on BMI calculated from both measured and self-reported weight and height at 17 years of age (TAAG 2), we calculated sex-specific BMI-for-age based on growth charts developed by the Centers for Disease Control and Prevention [26]. Overweight was defined as BMI-for-age ≥85th percentile, obesity as ≥95th percentile.

The primary study outcome for this analysis is the relative difference between self-reported and measured body weight at 17 years of age. The choice of the primary outcome was driven by our hypothesis that depression and body perception are associated with body weight and the accuracy of reporting it. We decided not to use BMI as an outcome because this measure also depends on the accuracy of self-reported height. For descriptive purposes (Table 1), we also classified girls as correct reporting if the relative difference between self-reported and measured body weight was within ±3% of the measured body weight. If the self-reported body weight deviated by more than 3%, girls were classified as under-reporters if the difference was negative and as over-reporters if the difference was positive. The cutoff point of 3% was chosen to allow a range of ~0.5 kg for a correctly reported weight.

As predictors for over- and underreporting, we also calculated the change in body weight and height between the ages of 11 and 17 years (delta body weight, delta height, respectively)

#### 2.2.2. Depression Scale

Depressive symptoms were measured using the Center for Epidemiological Studies-Depression Scale (CES-D), a 20-item questionnaire from which scores are calculated for depressive symptoms overall and for specific subscales [27,28]. For the present study, a scoring system developed by Carleton et al. was used [29]. This scoring system addresses the previously criticized sex difference in responses that leads to an inflation of females’ CES-D scores due to cultural norms regarding emotional expression, rather than actual differences in depressive symptoms (Carleton 19 and 20). The Carleton scoring system uses only 14 out of 20 items and calculates 3 (instead of 4) different factors (i.e., negative affect, anhedonia, and somatic symptoms) that are consistent with current diagnostic criteria for depression [29]. For the present study, the overall Carleton depression score was based on 14 items as well as 3 subscale factors used as potential predictors. The raw Cronbach’s alpha for the overall scale and the negative affect, anhedonia, and somatic symptoms subscales were 0.74, 0.82, 0.81 and 0.73, respectively.

#### 2.2.3. Physical Self Concept

For TAAG 2, participants completed the Physical Self-Description Questionnaire, a multidimensional physical self-concept instrument [30,31,32]. Three subscales were assessed: the 8-item global esteem scale, the 6-item global physical self-concept scale, and the 6-item body fat scale. The global esteem scale rates overall positive feelings about oneself, the global physical self-concept scale rates positive feelings about one’s physical self, higher scores indicate greater positive perception [32]. The body fat subscale rates the perceived body fat, a higher scale indicates a greater perception to not be overweight. The raw Cronbach’s alpha for the global self-esteem scale, the general physical self-concept scale, and the body fat scale were 0.96, 0.88 and 0.95, respectively.

#### 2.2.4. Race/Ethnicity and Other Individual-Level Factors

Girls self-identified as non-Hispanic white, black or African American, Hispanic or Latino, Asian/Pacific Islander, American Indian or Alaska Native or other were categorized as white, black, Hispanic, or other. The girls reported their participation in the subsidized school lunch program and parental education level as proxy for socioeconomic status.

### 2.3. Statistical Analysis

Differences in demographic and other factors were evaluated using the Kruskal–Wallis test and the chi-square test between girls who correctly reported their body weight and those who over- or underreported their body weight at age 17. Due to the distribution of some variables, median values of descriptive variables (Table 1) were compared by using the Kruskal–Wallis test and correlations were evaluated by Pearson correlation coefficients. To measure the internal consistency between overall and subscales in depression and the questions of depressive symptoms used to create the scales, Cronbach’s alpha were calculated.

To determine the limits of agreement, Bland–Altman plots show the difference between self-reported and measured weight, as well as BMI calculated from self-reported and measured weight and height (*y*-axis) shown as function of the arithmetic mean of self-reported and measured values (*x*-axis) [33]. The limits of agreement are given as 1.96 × SD of the difference. Pearson correlation and intraclass correlation coefficient (ICC) are provided for the self-reported and measured values of weight, height, and BMI.

To identify factors that explain the extent of the misreporting of body weight (defined as the relative difference between measured and self-reported weight at age 17 years), models were fit using multivariable linear regression analysis. First, the following potential demographic and other factors, coded and categorized as previously mentioned, were included in the full model: race/ethnicity (non-Hispanic white, black, Hispanic, other), participation in subsidized school lunch (yes/no/unknown), parental education level (high school or less, some college, college graduate or higher), parental employment status (both parents working full-time, one parent working full-time, other), BMI-for-age category (under/normal weight, overweight, obese), general physical self-concept scale, global self-esteem scale, body fat scale, overall depressive symptoms, negative affect, anhedonia, and somatic symptoms, measured height, difference between self-report and measured height, change in height between time 1 and 2 (Δ height) and change in body weight between time 1 and 2 (Δ weight). Using backward selection, we then removed non-significant independent factors with the lowest F statistics one by one until all remaining factors were significant at *p* < 0.10. Second, we added interaction terms of the remaining factors in the reduced model from the first step, then removed non-significant terms until all remaining terms were significant at *p* < 0.10. Models were then compared based on Akaike information criterion (AIC), corrected AIC (AIC_C_) for small sample size and Bayes information criterion (BIC)—the model with the lowest values was chosen. [34] No significant interactions were found. Due to the high correlation among subscales of depressive symptoms, we evaluated both overall score and subscales in separate models, due to weak associations between overall depression score and outcome, we only included subscales of depressive symptoms separately in the model. Estimates, standard errors (SE), *t* values and *p* values were reported for each of the terms in the final model. All analyses were performed using SAS statistical software version 9.4 (SAS Institute Inc, Cary, NC, USA).

## 3. Results

### 3.1. Results

#### 3.1.1. Description of the Study Population

Of the 588 participants, 279 (47.4%) were non-Hispanic whites, 126 (21.4%) were black, 78 (13.3%) were Hispanic, and the remainder were classified as “other” (17.9%, Table 1). The majority of girls (70.9%) had at least one parent with college education or higher, and about 21.3% participated in the free school lunch program (Table 1). At 17 years of age, 173 (29.4%) of girls were classified as overweight or obese based on measured weight and height (Table 1).

#### 3.1.2. Differences between Self-Reported and Measured Weight, Height and Body Mass Index

Self-reported body weight was classified as accurate for most girls. Using a relative difference of ±3% between self-reported and measured weight as cut-off, 59.1% of girls reported their weight correctly, 30.2% of girls underreported by an average of −5.8 ± 4.8 kg, and 10.5% of girls over-reported by an average of 4.3 ± 3.5 kg. Among girls who were under/normal weight, 261 (62.9%) were classified correctly, whereas 47 (52.2%) of girls who were overweight and 40 (48.2%) of girls who were obese were classified correctly.

**Figure 1:**
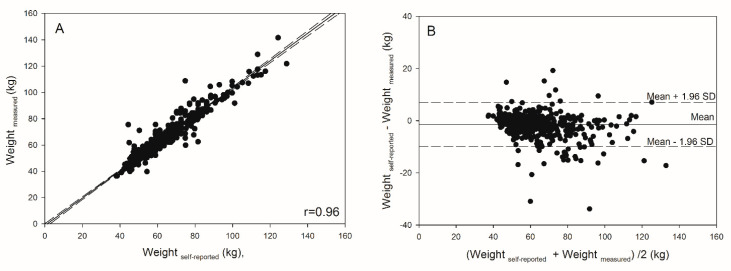
Linear regression (95% confidence interval) of self-reported and measured weight (**A**) and Bland–Altman agreement plots showing the difference between self-reported and measured weight (**B**) plotted against the arithmetic mean of both measurements in 17-year old girls (*n* = 588). A negative sign in the difference indicates an underestimation and a positive sign indicates an overestimation of self-reported weight.

The average difference between self-reported and measured body weight was −1.5 ± 4.3 kg (*p* < 0.001, Figure 1). The average difference between self-reported and measured height was 0.3 ± 2.5 cm (*p* = 0.99). The average difference between body mass index calculated from self-reported and from measured weight and height was −0.6 ± 1.8 kg (*p* < 0.001). Self-reported and measured body weight (r = 0.96, ICC = 0.95) and height (r = 0.93, ICC = 0.93) were highly correlated as were BMI calculated from self-reported and from measured weight and height (r = 0.94, ICC = 0.93).

We observed a dose–response relationship in the discrepancy between self-reported and measured weight with body weight (*p* < 0.0001 for weight). Figure 1B (Bland–Altman plot) illustrates the difference between self-reported and measured weight in relation to the mean of both values indicating that girls with higher weight tend to underreport while girls with lower weight or BMI tend to overreport.

#### 3.1.3. Predicting the Extent of Misreporting

Girls who were overweight and obese were more likely to underreport their body weight than girls who were normal or underweight. Girls who overreported their body weight were more likely to be under or normal weight, be less active and have a higher body fat and global physical self-concept scale as well as a higher negative affect (Table 1). Girls who underreported were more likely to be overweight, obese, and have lower scores for the body fat and global physical self-concept scale as well as a lower negative affect.

The final model to predicting the extent of the misreporting of body weight (defined as the relative difference between measured and self-reported weight and height) included overweight (β ± SE − 2.60 ± 0.66%), obesity (β ± SE − 2.41 ± 0.71%), weight change between ages 11 and 17 (β ± SE − 0.35 ± 0.04% for each kg of weight change), height change between ages 11 and 17 (β ± SE 0.29 ± 0.10% for each cm of height change), and negative affect (β ± SE 0.18 ± 0.08% for each score unit) (Table 2).

## 4. Discussion

While the difference between self-reported and measured body weight in adolescent girls is overall relatively small, about one out of three girls underreported and one out of ten girls overreported more than 3% of their body weight. The average difference between self-reported and measured body weight was −1.5 kg, which is within the range reported by other studies [1,10]. For a better understanding of factors predicting misreporting beyond overweight and obesity, the present study investigated several additional factors such as concept of physical self and depression. In adolescent girls, overweight, obesity, recent weight and height changes, as well as negative affect were associated with misreporting of body weight. Physical self-concept was not associated with misreporting after adjusting for body weight.

Despite a low average difference between self-reported and measured body weight, the misclassification of overweight or obese girls can be significant if a systematic reporting bias is observed. In a literature review, only between 59 and 70% of girls who were truly overweight or obese were classified as overweight or obese if self-reported weight and height was used to determine BMI [10]. A recent meta-analysis suggested that about 78% of US adolescents, independent of gender, would be classified correctly based on self-report [1], for adolescents who were obese, the proportion was slightly lower with 73% classified correctly based on self-report. In other words, self-reported data underestimated the prevalence of overweight and obesity. Between 20 to 40% of those overweight or obese would be missed when relying on self-report [1,10]. In the present study, about 40% of girls who were overweight and 23% of girls who were obese would be missed based on self-report.

For studies relying on self-report, a better understanding of which factors predict misreporting will improve the interpretability of future results. As observed in other studies [10,35,36,37,38], girls who participated in the present study and were overweight or obese were more likely to underreport their body weight. Rapid growth during adolescence and lack of knowledge about current weight may explain a lower accuracy of self-reported weight in adolescents. A low body weight response capability defined as longer time since last weighing or low ability to recall their body weight was associated with lower accuracy of self-reported weight [37]. However, in that study, longitudinal body weight was not available. The present study suggests that recent gain in body weight is a strong predictor of underreporting body weight while gain in body height is a predictor of overreporting body weight.

Among factors related to depressive symptoms and physical self-concept investigated in this study, negative affect was the only independent predictor of misreporting body weight. Negative affect assesses the strong experience of negative emotions such as anger, contempt, and anxiety. We were not able to identify other studies investigating the association between negative affect and misreporting of body weight. However, higher BMI was associated with lower body satisfaction, leading to higher negative affect in girls [39]. Higher frequency of body checking such as weighing, comparing one’s body to others, or checking body size on a reflective surface predicted higher body dissatisfaction and negative affect [40]. McCabe et al. also showed that low levels of body satisfaction, high levels of body image importance, and body change strategies such as dieting predicted high levels of negative affect and low levels of positive affect in girls [39]. In the present study, girls with a higher negative affect score were associated with overreporting after adjusting for body weight, recent changes in body weight and height, and other factors. We can only speculate if the overreporting of body weight observed in the present study is a disturbance of percept (i.e., distortion) as a result of negative affect leading to a low body satisfaction and resulting in a failure to evaluate the size of their body accurately [41]. This effect could be driven by a fear of negative evaluation as observed in girls with low self-esteem and high body mass index [42].

The present study contributes to our knowledge about the accuracy of self-reported body weight in girls. Several strengths should be noted. The longitudinal design of the study allowed us to identify recent changes in body weight as a strong predictor of misreporting body weight. A broad range of potential predictors including depressive symptoms, physical self-concept, and socioeconomic factors were available as potential predictors of misreporting. Another strength of the study was the measurement order (self-report occurred first) and the short time elapsed (minutes) between self-reported and measured body weight and height, which could contribute to measurement error and a bias towards the null. Girls participating in the study were also informed about the measurement of weight and height at the study visit, which could have led to a more accurate report of body weight and height than without this knowledge. Light clothes at the study visit may explain some of the difference between self-reported weight and measured weight at the study visit if individuals weighed at home without clothes. This could have led to a higher difference between self-reported and measured body weight. Other limitations include the limited age range of the population, which did not allow us to determine if the predictors of misreporting differ by age and may limit the generalizability to other age groups.

## 5. Conclusions

In conclusion, self-reported body weight in adolescent girls is relatively accurate but even these slight differences can lead to a significant underestimation of overweight and obesity when relying on self-reported body weight alone. Excess body weight and recent weight gain were associated with underreporting, while recent growth in height and negative affect were associated with overreporting of body weight. These factors should be taken into consideration when estimating measurement error in epidemiologic studies among adolescent girls relying on self-reported body weight.

## Figures and Tables

**Table 1:** Characteristics of girls at 17 years of age who underreported, correctly reported, or overreported body weight.

	All	Body Weight Reporting			*p*-Value		
		Under-Reported (>3%)	Correctly Reported (±3%)	Over-Reported (>3%)	Under vs. Correct	Over vs. Correct	Overall
***n***	588	178	348	62			
**Race/ethnicity (%)**					0.661	0.018	0.663
Non-Hispanic White	279 (47.4)	81 (45.5)	173 (49.7)	25 (40.3)			
Black/African American	126 (21.4)	40 (22.5)	64 (18.4)	22 (35.5)			
Hispanic	78 (13.2)	25 (14)	45 (12.9)	8 (12.9)			
Other	105 (17.9)	32 (18)	66 (19)	7 (11.3)			
**Free school lunch (%)**					0.432	0.015	0.333
No	444 (75.5)	134 (75.3)	272 (78.2)	38 (61.3)			
Yes	125 (21.2)	40 (22.5)	64 (18.4)	21 (33.9)			
Unknown	19 (3.2)	4 (2.2)	12 (3.4)	3 (4.8)			
**Highest education in parents (%)**					0.733	0.211	0.164
High school or less	171 (29.1)	49 (27.5)	100 (28.7)	22 (35.5)			
Some college	252 (42.8)	73 (41)	150 (43.1)	29 (46.8)			
College graduate or higher	165 (28)	56 (31.5)	98 (28.2)	11 (17.7)			
Body weight class (%) *					<0.001	0.009	<0.001
Normal/underweight	415 (70.5)	98 (55.0)	261 (75)	56 (90.3)			
Overweight	90 (15.3)	40 (22.5)	47 (13.5)	3 (4.8)			
Obese	83 (14.1)	40 (22.5)	40 (11.5)	3 (4.8)			
**Physical Self-concept**							
Body fat scale	4.0 (2.3; 4.8)	3.2 (2.0; 4.5)	4.0 (2.7; 4.8)	4.6 (4.2; 5.0)	0.003	<0.001	<0.001
Global physical self-concept scale	3.7 (2.5; 4.4)	3.1 (2.3; 4.3)	3.7 (2.6; 4.3)	4.3 (3.6; 4.6)	0.014	0.005	<0.001
Global esteem scale	4.3 (3.6; 4.6)	4.3 (3.5; 4.6)	4.2 (3.6; 4.6)	4.3 (3.8; 4.6)	0.965	0.733	0.519
Depressive symptoms							
Overall depression scale	11.0 (7.0; 17.0)	11 (7.0; 16.0)	11 (7.0; 17.0)	13 (8.0; 17.0)	0.434	0.421	0.277
Somatic symptoms scale	5.0 (3.0; 7.0)	5.0 (3.0; 7.0)	5.0 (3.0; 7.0)	6.0 (4.0; 7.0)	0.943	0.521	0.765
Negative affect scale	2.0 (0.0; 4.0)	1.0 (0.0; 4.0)	2.0 (0.0; 4.0)	3.0 (1.0; 5.0)	0.113	0.497	0.041
Anhedonia scale	4.0 (2.0; 6.0)	4.0 (2.0; 6.0)	4.0 (2.0; 6.0)	4.0 (2.0; 6.0)	0.678	0.946	0.798
**Body weight and height**							
Body weight (kg)							
Self-reported	58.7 (52.2; 68.0)	61.2 (53.1; 72.1)	58.1 (52.2; 65.8)	56.1 (47.6; 66.7)	0.027	0.156	0.012
Measured	59.5 (52.7; 68.8)	66.2 (57.1; 78.3)	58 (52.1; 66.7)	53.3 (45.0; 62.0)	<0.001	0.001	<0.001
Δ Self-reported-measured	−0.8 (−2.4; 0.5)	−4.1 (−6.9; 2.6)	−0.3 (−1.1; 0.5)	3.2 (2.1; 4.8)	<0.001	<0.001	<0.001
Δ Body weight, 11 to 17 years (kg) *	5.7 (2.6; 9.8)	8.5 (3.9; 13.7)	5.2 (2.4; 8.7)	3.0 (−1.0; 5.2)	<0.001	<0.001	<0.001
Height (cm)							
Self-reported	162.6 (157.5; 167.6)	162.6 (157.5; 167.6)	162.6 (157.5, 167.6)	161.3 (154.9, 167.6)	0.061	0.829	0.072
Measured	162.0 (158.0; 166.5)	162.3 (159.1; 167.3)	161.6 (157.3, 166.1)	161.3 (157.0, 167.0)	0.066	0.981	0.057
Δ Self-reported-measured	0.3 (−1.0; 1.9)	0.3 (−1.1; 1.8)	0.3 (−0.9, 1.9)	0.6 (−1.1, 1.7)	0.810	0.807	0.991
Δ Height, 11 to 17 years (cm) *	2.6 (1.6; 4.3)	2.5 (1.6; 4.3)	2.5 (1.7, 4.3)	2.7 (1.1, 3.9)	0.946	0.541	0.666
BMI (kg/m^2^)							
Self-reported	22.0 (19.9; 25.1)	22.7 (20.0; 26.5)	21.9 (20.0; 24.8)	21.3 (19.1; 25.1)	0.216	0.106	0.030
Measured	22.5 (20.4; 26.1)	24.5 (21.4; 29.3)	22.2 (20.3; 25.1)	20.1 (17.8; 22.8)	<0.001	<0.001	<0.001
Δ Self-reported-measured	−0.4 (−1.2; 0.3)	−1.8 (−2.8; −1.0)	−0.2 (−0.7; 0.3)	1.2 (0.7; 2.0)	<0.001	<0.001	<0.001
Δ BMI, 11 to 17 years *	1.4 (0.3; 2.8)	2.2 (1.0; 4.0)	1.3 (0.3; 2.4)	0.4 (−0.9; 1.4)	<0.001	0.009	<0.001

* Based on measured weight (and height). For consistency, median and interquartile range were reported for all continuous variables. Chi-square *p* value was used to test the equality of proportions for categorical variables. Kruskal–Wallis test *p*-value was reported for comparison of medians.

**Table 2:** Parameter estimates from linear regression model predicting the relative difference between measured and self-reported body weight in girls at age 17 (*n* = 588).

	Parameter Estimate β	SE	*t* Value	*p*-Value
Intercept	3.10	5.99	0.52	0.605
Race/ethnicity				0.404
White	0.0			
Black	1.03	0.61	1.70	0.090
Hispanic/Latina	0.25	0.64	0.39	0.696
Other	0.20	0.73	0.28	0.783
Height (cm) at 17 years	−0.02	0.04	−0.62	0.534
Body weight class at 17 years ^1^				<0.001
Normal/underweight	0.0			
Overweight	−2.60	0.66	−3.94	<0.001
Obese	−2.42	0.71	−3.42	<0.001
Δ Body weight (kg) ^2^	−0.35	0.04	−8.76	<0.001
Δ Height (cm) ^2^	0.29	0.10	2.83	0.005
Negative affect (scale units) ^3^	0.18	0.08	2.18	0.030

^1^ Calculated based on body mass index (BMI)-for-age from measured weight and height. ^2^ Δ Body weight/height between age 11 and 17 yrs. ^3^ Center for Epidemiologic Studies Depression Scale (CES-D) using a model according to Carleton et al. [29].

## Abbreviations

BMI	body mass index
KPSC	Kaiser Permanente Southern California
SE	Standard error
